# New Insights into Structural and Functional Roles of Indole-3-acetic acid (IAA): Changes in DNA Topology and Gene Expression in Bacteria

**DOI:** 10.3390/biom9100522

**Published:** 2019-09-23

**Authors:** Roberto Defez, Anna Valenti, Anna Andreozzi, Silvia Romano, Maria Ciaramella, Paolo Pesaresi, Sara Forlani, Carmen Bianco

**Affiliations:** 1 Istituto di Bioscienze e BioRisorse, via P. Castellino 111, 80131 Naples, Italy; roberto.defez@ibbr.cnr.it (R.D.); anna.valenti@ibbr.cnr.it (A.V.); anna.andreozzi@ibbr.cnr.it (A.A.); silvia.romano@ibbr.cnr.it (S.R.); maria.ciaramella@ibbr.cnr.it (M.C.); 2 Dipartimento di Bioscienze, Università degli Studi di Milano, via Celoria 26, 20133 Milan, Italy; paolo.pesaresi@unimi.it (P.P.); sara.forlani@unimi.it (S.F.)

**Keywords:** indole-3-acetic acid, DNA supercoiling, topoisomerases, gene expression, nitrogen-fixation genes

## Abstract

Indole-3-acetic acid (IAA) is a major plant hormone that affects many cellular processes in plants, bacteria, yeast, and human cells through still unknown mechanisms. In this study, we demonstrated that the IAA-treatment of two unrelated bacteria, the *Ensifer meliloti* 1021 and *Escherichia coli,* harboring two different host range plasmids, influences the supercoiled state of the two plasmid DNAs in vivo. Results obtained from in vitro assays show that IAA interacts with DNA, leading to DNA conformational changes commonly induced by intercalating agents. We provide evidence that IAA inhibits the activity of the type IA topoisomerase, which regulates the DNA topological state in bacteria, through the relaxation of the negative supercoiled DNA. In addition, we demonstrate that the treatment of *E. meliloti* cells with IAA induces the expression of some genes, including the ones related to nitrogen fixation. In contrast, these genes were significantly repressed by the treatment with novobiocin, which reduces the DNA supercoiling in bacterial cells. Taking into account the overall results reported, we hypothesize that the IAA action and the DNA structure/function might be correlated and involved in the regulation of gene expression. This work points out that checking whether IAA influences the DNA topology under physiological conditions could be a useful strategy to clarify the mechanism of action of this hormone, not only in plants but also in other unrelated organisms.

## 1. Introduction

Auxin biology is among the oldest fields of experimental plant research. Back in 1880, it was proposed [1], for the first time, that a diffusible molecule, sensitive to light, was transported downward and bent coleoptiles towards light. Later on, Went and Thiemann [2] described this factor and identified the molecule as being indole-3-acetic acid (IAA). Nearly eight decades after the structural elucidation of IAA, many aspects of auxin metabolism, transport, and signaling have been well established; nevertheless, more than a few fundamental questions and innumerable details remain unsolved. Furthermore, in the last two decades, it has been reported that IAA induces major changes in developmental and metabolic functions in quite diverse systems, such as cancer cells [3], plants [4], fungi [5], and bacteria [6,7,8], through still unknown mechanisms.

We previously reported that the treatment of *Escherichia coli* cells with purified IAA induced the expression of genes involved in energy metabolism pathways and led to an improved stress response [6,7]. Furthermore, the overproduction of IAA in the strain *Ensifer meliloti* RD64, a derivative of the *E. meliloti* 1021 engineered to overproduce the auxin indole-3-acetic acid (IAA) [8,9], directly or indirectly led to the activation of genes involved in nitrogen-fixation and energy metabolism processes under free-living conditions [10,11,12]. The induction of nitrogen-fixation genes in response to increased IAA availability was also observed in diazotrophic bacteria associated with cereals [13,14]. These results lead us to speculate that the activation of nitrogen-fixation (*nif* and *fix*) genes expression triggered by IAA is a widespread effect. To shed light on the mechanisms through which IAA influences the expression of certain genes, and in particular the ones involved in nitrogen-fixation, we considered that: (1) The activation of *nif* and *fix* genes’ expression in *E. meliloti* is mediated by the transcriptional activator *nifA* [15]; and (2) the *nifH* promoter activity in *E. meliloti* is stimulated by changes in DNA supercoiling [16]. The data published some 40 years ago by Witham et al. [17] and Jacobsen [18] were our starting point. These authors hypothesized possible in vivo stereochemical recognitions between nucleic acids and intercalated phytohormones. It is well established that the DNA binding of external ligands transiently alters the local topology of DNA, inducing variations in its structure. Moreover, the negative supercoiling is essential for optimal functioning of the transcription processes, as it facilitates promoter melting [19,20,21]. It is also known that maintenance of the steady-state level of negative DNA supercoiling in bacteria relies largely on the supercoiling action of DNA gyrase and the relaxation activity of DNA topoisomerase I [22]. Type IA DNA topoisomerase I is a ubiquitous enzyme present in all bacteria. The inhibition of gyrase activity in vivo by a specific inhibitor (i.e., novobiocin) alters the supercoiled state of DNA, with a negative impact on cell viability.

By using in vivo and in vitro assays, we here verify, for the first time, that IAA: (i) Influences the DNA topological state in bacteria; (ii) interacts directly with DNA in vitro; (iii) inhibits the DNA relaxation activity of the bacterial type IA topoisomerase; (iv) induces the expression of bacterial genes sensible to DNA topological changes; and (v) activates the promoter of *nifA* gene, the main regulator of nitrogen-fixation genes. A further deepening of our results could help to understand whether IAA plays direct and/or indirect roles in modulating the DNA topology, and what could be the biological implications on the regulation of gene expression.

## 2. Materials and Methods

### 2.1. Bacterial Strains, Growth Conditions, and Plasmids

The bacterial strains used in this study were: The *Ensifer meliloti* 1021 [23]; *E. meliloti* 1021 harboring the plasmid pMB393 [6]; *E. meliloti* 1021 harboring the recombinant plasmid pCHK57 [24]; and *E. coli* BL21 harboring the reporter plasmid pBluScript (pBS). *E. meliloti* 1021 competent cells’ preparation and transformation with the plasmid pMB393 were carried out as previously described [6]. BL21 competent cells’ preparation and transformation with the plasmid pBS were carried out according to standard protocols. For the analysis of β-galactosidase activity, the *E. meliloti* 1021 cells harboring the *nifA*-*lacZ* translational fusion plasmid pCHK57 and the *E. meliloti* 1021 wild type ones were grown aerobically at 30 °C in TYR medium (5 g L^−1^ tryptone, 3 g L^−1^ yeast extract, 6 mM CaCl_2_) for 48 h, washed three times with nitrogen-free RMM minimal medium, and then re-suspended in the same medium supplemented with 5 mM glutamate and appropriate antibiotics. Bacterial cultures (OD_600_ = 0.6) were then split into four aliquots: An IAA solution was added to one culture to a final concentration of 1.0 mM, to the second and third ones a novobiocin solution was added to the final concentrations of 15 and 1.5 mM, and the fourth one was left untreated (control). After 0.5, 1.0, 1.5, 3, and 6 h of incubation at 30 °C, independent cell batches of control and treated cells were collected and immediately used for the β-galactosidase activity assay as described by Miller [25]. The IAA and novobiocin stock solutions were prepared using a 50% (*v*/*v*) dimethyl sulfoxide (DMSO) solution. To avoid solvent interference, control cells were treated with a similar amount of DMSO solution.

To analyze the effect induced by IAA and novobiocin treatment on gene expression by qRT-PCR analysis, *E. meliloti* 1021 harboring the pMB393 plasmid were grown up to the exponential phase in RMM-glutamate medium as described above and subjected to a 1-hour treatment with IAA 1 mM or novobiocin 15 μM. Preliminary experiments were carried out to select the concentration of novobiocin to be used to analyze its effect on the expression of selected genes, without having a negative impact on the synthesis of both DNA and RNA [26]. We verified that the best novobiocin concentration was 15 μM. Both the untreated and treated cells were then collected, and the RNA was isolated as described by Imperlini et al. [11].

### 2.2. Evaluation of DNA Supercoiling In Vivo

In vivo supercoiling was monitored by determining the relative topoisomer distribution of the reporter plasmids pMB393 (*E. meliloti* 1021) and pBS (*E. coli* BL21). *E. meliloti* 1021 bacterial cells were grown in RMM-glutamate medium up to the exponential phase and then subjected to 0.5-, 1.0-, and 1.5-h treatments with IAA (0.2, 1, and 5 mM) or novobiocin (15 and 75 μM) as described above. pMB393 DNA plasmid was purified from *E. meliloti* 1021 cells by the QIAprep Spin Miniprep Kit (QIAGEN, Hilden, Germany) according to the manufacturer’s instructions. *E. coli* BL21 cells were grown in M9 minimal medium [6] until the exponential phase (OD_600_ = 0.6), and then treated with IAA (0.2, 1, and 5 mM) or novobiocin (15 and 75 μM) for 0.5, 1.0, and 1.5 h. pBS DNA plasmid was purified from *E. coli* BL21 cells by the NucleoSpin Plasmid kit (Macherey Nagel, Duren, Germany) according to the manufacturer’s instructions. The DNA topology of purified plasmids were analyzed as described below. Topoisomers were separated by electrophoresis in 0.7% agarose gels containing chloroquine to the final concentrations of 1 (pBS) or 10 μg mL^−1^ (pMB393). Electrophoresis was carried out in Tris-borate-EDTA (TBE) buffer 0.5X containing 1 or 10 μg mL^−1^ chloroquine at 100 V (4.5 V/cm) for 20 h at room temperature. After electrophoresis, chloroquine was washed from the gel by soaking in distilled water for 2 h (gels containing 1 μg mL^−1^ chloroquine) or 24 h (gels containing 10 μg mL^−1^ chloroquine) before staining with ethidium bromide (EtBr) (1 μg mL^−1^). Finally, the gel was washed in distilled water and photographed under UV light using the VersaDoc imaging system (Bio-Rad, Hercules, CA, USA). The relative intensity of each band was measured by densitometry with the Quantity One software (Bio-Rad, Hercules, CA, USA) and used to calculate the total amount of DNA and the relative abundance of each topoisomer. Topological properties of DNA were defined by specific parameters: Twist (T_w_), the number of times each helix twists around the other, and writhe (W_r_), the number of time the axis of the helix crosses itself. In a covalently closed DNA molecule, the sum of these two parameters is a fixed topological property, called the linking number (L_k_ = T_w_ + W_r_) [27]. Because the energy required to supercoil a polymer depends on its length, a useful parameter to compare different topological isomers (topoisomers) is the supercoiling density, σ = (L_k_ − L_k0_)/L_k0_, where *Lk^0^* and *Lk* represent the DNA linking numbers for the relaxed and supercoiled DNA, respectively. The superhelical density of topoisomers with different supercoiling degrees was estimated by one-dimensional electrophoresis in the presence of chloroquine as an intercalating agent, which causes a decrease in Tw, and consequently an increase of Wr. Under these conditions, the highly negative plasmid migrate as different bands with a specific ΔLk. The DNA linking number of each topoisomer was determined and used to obtain σ values applying the above equation. The relative amount of all topoisomers was expressed as a percentage of single topoisomer with respect to the total DNA in each lane [28].

### 2.3. DNA Binding Experiments

#### 2.3.1. Absorption Spectra Titration

UV absorption spectra of IAA were performed on a Cary 50 spectrophotometer (Agilent, Santa Clara, CA, USA) using 1.0-cm quartz cuvettes. Absorbance titrations were employed keeping the concentration of IAA constant and varying the DNA one. An IAA stock solution was prepared at a final concentration of 25 μM in 50% ethanol buffer and incubated with different amount of pBS (0.2, 0.4, 0.8, and 1.7 μg mL^−1^). The spectroscopic measurements were recorded after five minutes to allow the binding equilibrium between the IAA and DNA. The spectra were recorded at room temperature in the wavelength range of 200 to 350 nm at regular steps of 0.15 nm and a 60 nm min^−1^ scan rate. Appropriate mixtures of ethanol buffer and pBS plasmid were used as a reference and subtracted from each measurement.

#### 2.3.2. Competitive Study with EtBr

A competitive EtBr binding assay was carried out to investigate the interaction of ctDNA with IAA by using the UV-Visible (UV-Vis) spectroscopy. UV-Vis absorption spectra were obtained on a Cary 100 spectrophotometer (Agilent, Santa Clara, CA, USA) using 1.0 cm quartz cuvettes at room temperature. The stock concentration of ctDNA was determined using the molar extinction coefficient values of 6600 M^−1^ cm^−1^ at 260 nm. The absorption spectra of EtBr (7 μM) were recorded in the wavelengths range of 400 to 600 nm in the absence and presence of ctDNA (300 μM). The absorption spectra of the EtBr–ctDNA complex in the presence of increasing concentrations of IAA (from 2 μM to 25 mM) were also measured.

#### 2.3.3. Circular Dichroism (CD) Analysis

Circular dichroism (CD) measurements were performed on a Jasco J-810 spectropolarimeter (Jasco, Easton, MD, USA) using a 0.1 cm path-length quartz cuvette at room temperature and the following parameters: Three scans at a speed of 20 nm min^−1^, 4 s time constant. The spectral bandwidth was set up at 1 nm in the wavelength range of 225 to 300 nm. The CD spectra of 125 ng mL^−1^ ctDNA were measured under a nitrogen atmosphere using a 10 mM Tris HCl buffer at pH 7.5 and increasing concentration of IAA (0.0315, 0.0625, 0.125, 0.25, and 0.5 mM). Results are presented as a mean of three scans and the buffer background was electronically subtracted from each measurement.

### 2.4. Topoisomerase-Mediated DNA Relaxation Assay

DNA relaxation assays were performed using the pBS plasmid, purified from *E. coli* cultures in its negatively supercoiled form, as a substrate of DNA topoisomerases from *E. coli*. To test the effect of IAA on topoisomerase activity, IAA stock solutions were prepared in 50% (*v*/*v*) ethanol and added to DNA plasmid (7.0 μg mL^−1^). To avoid solvent interference, control reactions without IAA were incubated in the same conditions with an equal volume of ethanol solution. The mixtures were incubated for 15 min at RT to ensure binding equilibrium. The topoisomerase was then added to the reaction mixture to achieve relaxation of the DNA according to the manufacturer’s instruction. The reaction mixture contained 50 mM potassium-acetate, 20 mM Tris-acetate, 10 mM Magnesium-acetate, and 100 μg mL^−1^ BSA (pH 7.5). Preliminary experiments were carried out using topoisomerase I from *E. coli* (*EcTopoI*) (New England BioLabs, Evry, France) and graded concentrations of IAA (0.2, 1.0, and 3 mM) to select the best one. The *EcTopoI* enzyme has a 40% amino acid sequence homology to the *E. meliloti* topoisomerase I. The 1 mM concentration of IAA was chosen for subsequent experiments. The concentrated stock of the *EcTopoI* was serially diluted and added to the reaction mixtures (0.1, 0.5, and 2.5 U mL^−1^), and then incubated at 37 °C for 45 min. To verify the specificity of IAA, purified molecules, structurally (indole, IND; indole-3-carboxylic acid, ICA; tryptophan, Trp) or functionally (2,4-dichlorophenoxyacetic acid, 2,4-D) similar to IAA, were also tested at a final concentration of 1 mM using *EcTopoI* (2.5 and 5 U mL^−1^). All the reactions were stopped by the addition of SDS to a final concentration of 1% and the products were analyzed by electrophoresis on a 1.2% agarose gel in the presence of 50 mM Tris-Borate EDTA buffer. Supercoiled DNA plasmids were loaded at the same time as a control of plasmid migration. To visualize the reaction products, the gel was stained post-electrophoresis by soaking in a bath containing ethidium bromide (1 μg mL^−1^) for 30 min at room temperature and destained in deionized water for at least 1 h. Finally, the gel was imaged with UV light by a Gel-Doc Imaging System (UVP, Upland, CA, USA), and analyzed by the Image Lab Software (Bio-Rad, Hercules, CA, USA). The relative intensity of each band in the gel was measured and used to calculate the amount of total DNA (the unprocessed DNA plus the distinct topoisomer species), and that of all individual reaction products. For each condition, the topoisomerase I activity was expressed as a percentage and calculated by dividing the intensity of each topoisomer band by the amount of total DNA in each lane.

### 2.5. DNA Gyrase Assay

DNA gyrase assays were performed using a relaxed DNA plasmid as a substrate of the gyrase topoisomerase from *E. coli* (*EcGyr*, New England BioLabs, Evry, France). Briefly, relaxed DNA was obtained by incubating the negative supercoiled pBS with the topoisomerase I from wheat germ (Promega, Madison, WI, USA), accordingly to the manufacturer’s instruction. After incubation, the plasmid was purified by phenol extraction and ethanol precipitation and used for the gyrase assay. Before the addition of the enzyme, the DNA plasmid (1.9 μg mL^−1^) was incubated with or without IAA (1 mM) for 15 min at room temperature to ensure binding equilibrium. Increasing amounts of gyrase (75, 150, 375, and 500 U mL^−1^) were then added to the mixture and incubated at 37 °C for 30 min. The reactions were terminated by the addition of 1% SDS and analyzed by agarose gel electrophoresis as described before. Linearized DNA plasmid was loaded as a control of plasmid migration.

### 2.6. Quantitative Real-Time PCR (qRT-PCR) Analysis

The isolation of RNA from control, IAA-treated, and novobiocin-treated cells was carried out as described by Imperlini et al. [11]. Residual DNA present in the RNA preparations was removed by using the RNAse-free TURBO DNase I Kit (Applied Biosystems, Waltham, MA, USA) according to the manufacturer’s instructions. After purification and quality checking by agarose gel electrophoresis, the RNA concentration was determined by absorbance at 260 nm and the RNA was stored at −20 °C until further use. First-strand cDNA was synthesized from 1 µg of total RNA with the RETROscript kit (Applied Biosystems, Waltham, MA, USA) and random decamers, according to the manufacturer’s instructions. qRT-PCR was performed as previously described [29]. Specific primer pairs used for this analysis were designed using Primer3 software and are reported in Table 1. Primers for *rpoB* [9] were included in all the qRT-PCR analyses for the purpose of data normalization. During the reactions, the fluorescence signal due to SYBR Green intercalation was monitored to quantify the double-stranded DNA products formed in each PCR cycle. Results were recorded as relative gene expression changes after normalizing for *rpoB* genes expression, and computed using the comparative CT method (2^–^^ΔΔCT^) as previously described in Livak and Schmittgen [30]. The 2^–^^ΔΔCT^ value was >1 for genes more highly expressed in IAA- or novobiocin-treated 1021 cells and <1 for genes more highly expressed in untreated 1021 cells. qRT-PCR data are the mean ± standard deviation (SD) of at least four biological replicates each conducted at different times.

## 3. Results

### 3.1. IAA Influences DNA Supercoiling In Vivo

To verify whether IAA affected the DNA topology in vivo, the level of DNA supercoiling was evaluated in two unrelated bacteria: The soil bacterium and poor IAA-producer *E. meliloti* 1021 and the enteric bacterium *E. coli*, which does not synthesize IAA and for which we have already demonstrated that IAA plays a role in gene expression changes [7]. The *E. meliloti* and *E. coli* cells harboring the pMB393 (7.000 bp) and pBS (3.000 bp) plasmids, respectively, were treated over time with graded concentrations of IAA. Plasmid DNA was purified from untreated (control) and IAA-treated cells and electrophoresed in the presence of chloroquine.

**Figure 1:**
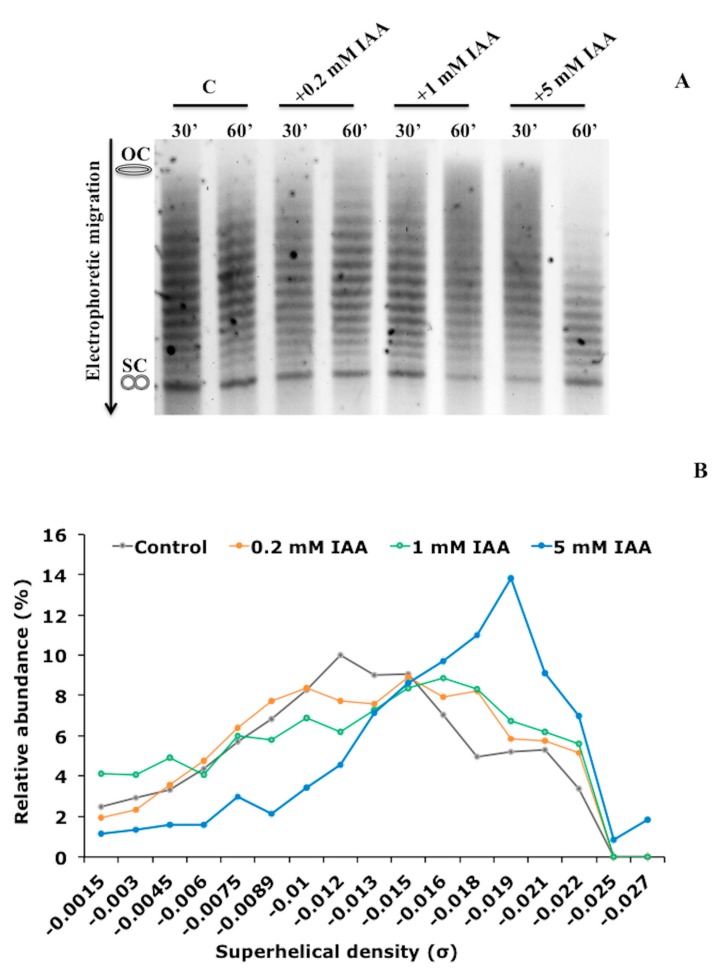
Effect of indole-3-acetic acid (IAA) on DNA topology in *Ensifer meliloti* cells. (**A**) Analysis of the relative topoisomers distribution for the plasmid pMB393. The pMB393 plasmid was purified from *E. meliloti* 1021 control cells and the ones treated for 30 and 60 min with IAA (0.2, 1, and 5 mM) and separated by electrophoresis in 0.7% agarose gels containing 10 μg mL^−1^ chloroquine. (**B**) Topoisomers’ distribution calculated as a percentage of topoisomers with specific superhelical density versus total DNA in untreated control cells, and cells treated for 60 min with 0.2, 1, and 5 mM IAA. The topological state of each topoisomer is expressed as the specific superelical density (σ). OC, open-circular DNA; SC, supercoiled DNA. The electrophoretic mobility reported in Figure 1A is a representative image of three biological replicates.

**Figure 2:**
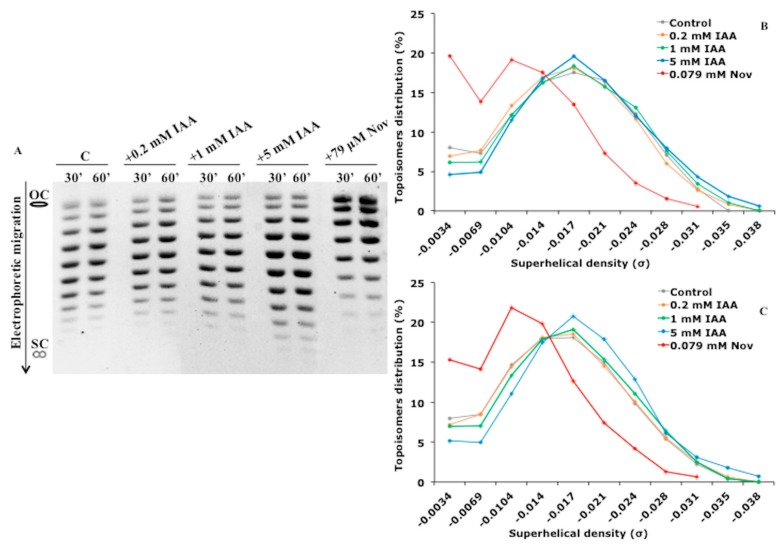
Effect of IAA on DNA topology in *Escherichia coli* cells. (**A**) Analysis of the relative topoisomers distribution of the reporter plasmid pBlueScript (pBS). The pBS plasmid was purified from *E. coli* BL21 control cells and the ones treated for 30 and 60 min with IAA (0.2, 1, and 5 mM) or novobiocin (79 µM) and separated by electrophoresis in 0.7% agarose gels containing 1 μg mL^−1^ chloroquine. (**B** and **C**) Topoisomers’ distribution calculated as a percentage of topoisomers with specific superhelical density versus total DNA in each condition after 30 and 60 min of treatment, respectively. The topological state of each topoisomer is expressed as the specific superhelical density (σ). OC, open-circular DNA; SC, supercoiled DNA. The electrophoretic mobility reported in Figure 2A is a representative image of three biological replicates. Nov, novobiocin.

Figure 1A shows the electrophoretic mobility of plasmids extracted from *E. meliloti* cells treated with IAA for 30 and 60 min. The densitometry plots of plasmids extracted from *E. meliloti* cells treated with IAA for 60 min are also reported (Figure 1A,B and Figure S1). When 0.2 mM IAA was used, the overall pattern of topoisomers remained unchanged. However, the relative amount of each topoisomer changed as the concentration of IAA increased. In particular, the topoisomers having a high density of negative supercoiling (lower bands) were clearly visible after treatment with 5 mM IAA. Similar results were obtained for *E. coli*: The topoisomers generated after IAA treatment became more negatively supercoiled with increasing concentration of IAA (Figure 2A–C and Figure S2). By contrast, the in vivo treatment of *E. coli* cells with novobiocin inhibits the DNA gyrase activity and leads, as expected, to a reduction in the negative supercoiling level of plasmids [31] (Figure 2A–C). These results show that IAA increases the level of negative supercoiling in vivo in two unrelated bacteria, the soil bacterium and poor IAA-producer *E. meliloti* and the enteric bacterium *E. coli*, which does not synthesize IAA, suggesting that IAA influences the DNA topological state in vivo.

### 3.2. IAA Interacts with DNA Inducing Alteration in Its Structure

To assess whether IAA could interact directly with DNA, UV-Vis spectrophotometry and the circular dichroism (CD) were used [32,33,34].

#### 3.2.1. Absorption Spectra of IAA in the Presence of Plasmid DNA

**Figure 3:**
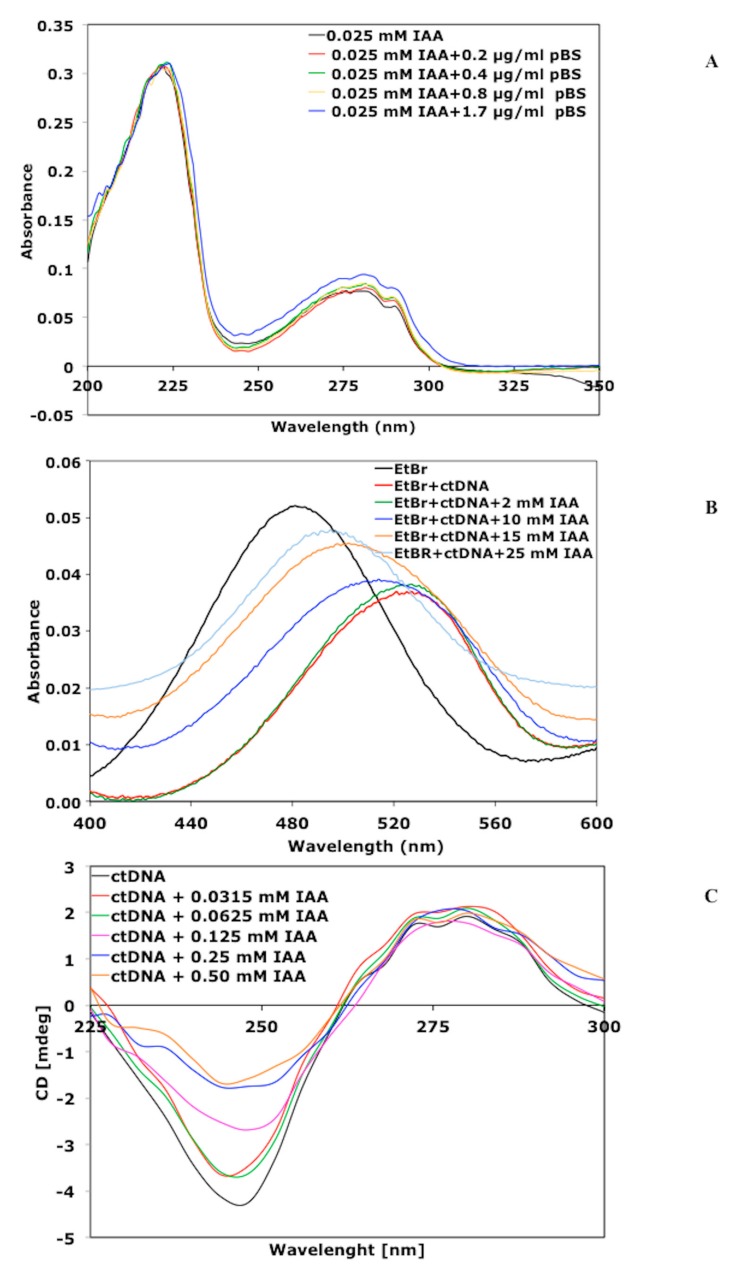
IAA alters the DNA structure. (**A**) UV absorption spectra of IAA (25 μM) with increasing amounts of pBlueScript (pBS) plasmid. Appropriate mixtures of ethanol buffer and pBS plasmid were used as reference and subtracted from each measurement. (**B**) UV-Vis absorbance spectra of ethidium bromide (EtBr) (7 μM) in absence (black line) and presence (blue line) of calf thymus DNA (ctDNA). The absorption spectra of the EtBr–ctDNA complex in the presence of increasing concentrations of IAA (2, 10, 15, 25 mM) were also reported. (**C**) Circular dichroism spectra of ctDNA (125 ng mL^−1^) in the presence of increasing concentrations of IAA.

We verified that the UV spectra of IAA were characterized by two maximum absorbance bands recorded at 220 and 280 nm [35,36,37]. When the pBS was added to the IAA solution at a final concentration of 1.7 μg mL^−1^, weak but reproducible hyperchromic changes in the absorbance at 280 nm were measured, probably as a consequence of the DNA–IAA complex formation (Figure 3A).

#### 3.2.2. Competitive Studies with Ethidium Bromide (EtBr)

The absorption spectrum of EtBr in aqueous solution is reported in Figure 3B. The addition of ctDNA led to a decrease in the absorbance of EtBr and to a red shift of its maximum absorption from 480 to 521 nm. The marked red shift is consistent with previous literature data [38,39], and is due to a decrease in the polarity of the environment of EtBr in the EtBr–ctDNA complex as compared to free EtBr. The addition of increasing concentrations of IAA to the EtBr–ctDNA complex led to a gradual increase (blue shift) of the absorbance and a new shift of the maximum absorbance from 521 to 489 nm.

#### 3.2.3. Circular dichroism (CD) Spectroscopic Analysis

The interaction between external molecules and DNA leads to significant changes in its structure and therefore to perturbations in its CD spectra [34,40]. The effect of IAA on the conformation of the secondary structure of ctDNA was investigated by CD study. Keeping the concentration of ctDNA fixed at 0.125 mg mL^−1^, the IAA concentration varied from 0 to 0.5 mM. As shown in Figure 3C the uncomplexed ctDNA in the B conformation displayed two CD bands in the UV region: A positive signal at 278 nm, due to base stacking interactions, and a negative band at 245 nm, characteristic of ellipticity. In the same wavelength range, the IAA alone did not display any CD spectra. From the CD study, it was found that the negative band decreased upon the addition of 0.0315 mM IAA, and decreased further as the concentration of IAA increased (Figure 3C). Such CD spectral changes clearly indicate that the binding of IAA with ctDNA led to a perturbation in the conformation of its secondary structure.

### 3.3. IAA Affects the Relaxation Activity of E. coli DNA Topoisomerases

**Figure 4:**
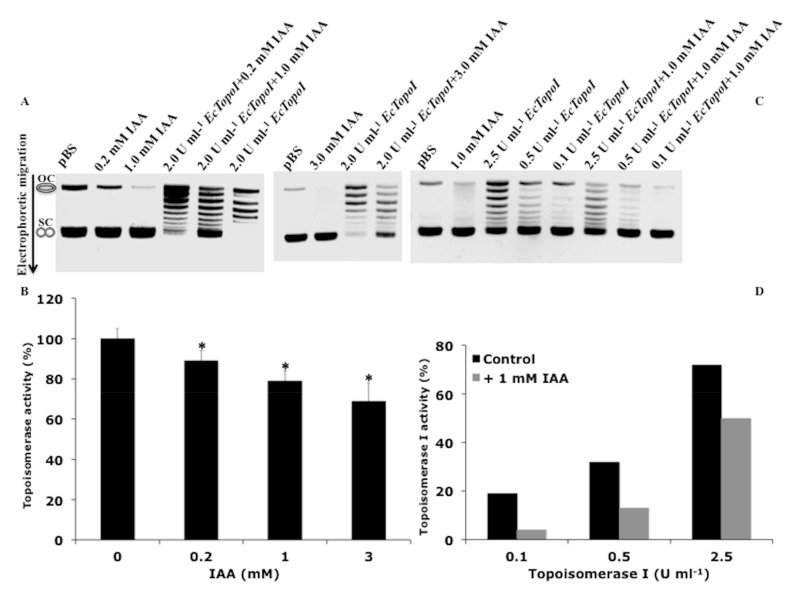
IAA influences the topoisomerase I activity. (**A**) and (**B**) Effect of 0.2, 1.0, and 3.0 mM IAA on the activity of 2.5 U mL^−1^ topoisomerase I from *E. coli* (*EcTopoI*). (**C**) and (**D**) Effect of 1.0 mM IAA on the activity of 0.1, 0.5, and 2.5 U mL^−1^ *EcTopoI*. To visualize the reaction products, the gels were stained post-electrophoresis with ethidium bromide and de-stained in deionized water. The relative intensity of each band in the gel was measured and used to calculate the amount of total DNA (the unprocessed DNA plus the distinct topoisomer species), and that of all individual reaction products. For each condition, the relaxation activity is expressed as a percentage of the produced topoisomers over the total DNA in each lane. Values are the means ± SD of three different biological replicates. The asterisks (*) indicate significant differences (*p* < 0.05, one-way ANOVA with Tukey’s post-hoc test) between the control and IAA-treated DNA samples. OC, open-circular DNA; SC, supercoiled DNA.

The topological state of DNA is tightly regulated in the cell by the combined activities of topoisomerases. DNA topoisomerases of different types have been found in organisms from all domains of life and play a key role in the resolution of critical topological problems in the DNA metabolism [41,42]. The supercoiling action of DNA gyrase and the relaxation action of DNA topoisomerase I play a main role in maintaining steady-state DNA supercoiling in bacteria. To further investigate the relationship between IAA action and DNA topology, we analyzed the effect of this molecule on the catalytic activity of DNA topoisomerases. The relaxation activity of topoisomerase IA from *E. coli* (*EcTopoI*) in the presence of IAA was assayed by exploiting the different electrophoretic mobility of the relaxed DNA molecules (topoisomers), as compared to the negatively supercoiled substrate. As shown in Figure 4A,C, the IAA alone did not affect the migration or the staining of the DNA substrate with a high supercoiling level (SC). On the contrary, a lower intensity of the open-circular (OC) DNA was observed after IAA treatment. The OC DNA is less stable than the CS DNA, thus the binding of IAA could further destabilize this DNA, making it more sensitive to degradation. When the DNA substrate was pre-incubated with IAA, and the *EcTopoI* then added to the reaction mixture, the relaxation activity of the enzyme was significantly inhibited in a concentration-dependent manner as compared to the untreated DNA (Figure 4A,B). Moreover, the IAA-mediated inhibition occurred at all concentrations of topoisomerase tested (Figure 4C,D), with an inhibition of 60% reached when 0.5 U mL^−1^ of *EcTopoI* and 1 mM IAA were tested as compared to the untreated DNA (Figure 4D). We verified that IAA exerted its inhibitor effect only when it was pre-incubated with the DNA substrate, and the *EcTopoI* was added later, while the simultaneous addition had no effect. These results indicate that: (1) The binding of IAA to DNA inhibits the relaxation activity of *EcTopoI*; and (2) IAA did not affect protein stability/function. To verify the specificity of the IAA inhibition, the *EcTopoI* activity was also tested in the presence of different molecules that were structurally and functionally similar to IAA. In our conditions, none of the compounds tested affected the relaxation activity of *EcTopoI*, indicating that the inhibition of topoisomerase activity is a peculiarity of IAA (Figure S3). In contrast, under the analyzed conditions, IAA did not exert a significant inhibiting effect on the supercoiling activity of the type II DNA gyrase, at all concentrations of enzyme tested (Figure S4).

### 3.4. IAA Triggers the Expression of Genes Sensitive to a DNA Gyrase Inhibitor

It is known that DNA supercoiling functions as a regulator of prokaryotic transcription [43,44]. Indeed, some promoters have an optimum level of supercoiling for their transcription, and this level is different for different promoters. Genetic studies have also shown that the superhelical state of cellular DNA in prokaryotes appears to be under equilibrium between the two major topoisomerases actions: DNA gyrase and topoisomerase I. We have previously reported [8] that IAA induced changes in the transcription of specific genes, including the ones related to nitrogen fixation (*nifA*, *fixK1*, *fixJ*), stress response (*dnaK*, *ibpa*, *rpoH1*), and metabolism (*pgi*, *sucA*, *sucD*, *icd*, *aceA*, *atpF2*). In this work, taking into account these data, we evaluated whether the expression of the selected group of genes was sensitive to specific changes in DNA topology. For this purpose, *E. meliloti* cells harboring the plasmid pMB393 [10] were treated with IAA, which inhibits topoisomerase activity, and novobiocin, which reduces the DNA supercoiling levels through inhibition of DNA gyrase. The expression level of the selected genes was measured by qRT-PCR analysis to check whether they were sensitive to specific DNA topological changes.

**Figure 5:**
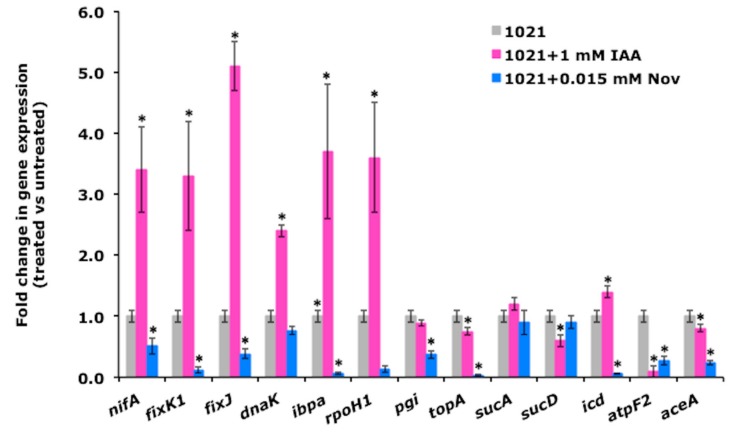
Alterations in DNA supercoiling mediated by IAA stimulate the expression of genes involved in nitrogen-fixation and stress response. Quantitative measurement of the *nifA, fixK1, fixJ, dnaK, ibpa, rpoH1, pgi,* and *topA* transcript levels in novobiocin- and IAA-treated cultures after 2 h of treatment. Values are the means ± SD of four different biological replicates. The asterisks (*) indicate significant differences (*p* < 0.05, one-way ANOVA with Tukey’s post-hoc test) between the control and treated cells. Nov, novobiocin.

Consistent with our previous data, we found that seven genes (*nifA*, *fixK1*, *fixJ*, *dnaK*, *ibpa*, *rpoH1*, and *icd*) were significantly induced in IAA-treated cells. For these genes, an opposite result was observed for the novobiocin-treatment: The transcript levels of almost all genes were strongly reduced, with a weaker repressive effect observed only for *dnaK* (Figure 5). The expression levels measured for the remaining genes showed that novobiocin did not repress all genes and that IAA did not induce any. These results suggest a possible correlation between the expression of the selected genes and the modifications observed in DNA topology.

### 3.5. nifA Promoter Activity is Stimulated by IAA and Inhibited by DNA Gyrase Inhibitor

**Figure 6:**
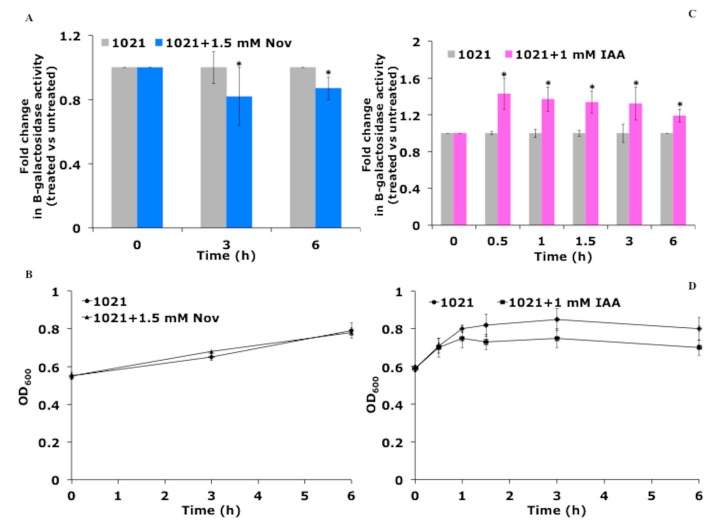
IAA stimulates the activity of the nitrogen-fixation regulator gene *nifA*. *E. meliloti* cells grown in nitrogen-free cultures were treated with IAA and novobiocin at final concentrations of 1 and 1.5 mM, respectively. The expression of the *nifA* promoter-lacZ fusion gene (**A** and **C**) and bacterial growth (**B** and **D**) were measured over time. The asterisks (*) indicate significant differences (*p* < 0.05, one-way ANOVA with Tukey’s post-hoc test) between the control and cells treated with IAA and novobiocin (Nov).

The *nifA* gene is the main transcriptional regulator involved in the expression of nitrogen fixation genes in *E. meliloti* [15]. In addition, Liu et al. [16] demonstrated that the gene expression driven by the *E. meliloti nifH* promoter required the presence of active DNA gyrase. Indeed, *nifH* promoter activity was significantly reduced when cells were treated with the DNA gyrase inhibitor novobiocin. Considering that DNA gyrase has the ability to introduce negative supercoils into DNA at the expense of adenosine triphosphate (ATP) hydrolysis, their results indicated that negative supercoiling was important for *nifH* promoter activity. Considering the ability of IAA to induce the expression of the *nifA* gene, we investigated whether IAA could also have an effect on the activity of the *nifA* promoter. Preliminary experiments were carried out to select the IAA and novobiocin concentrations that did not affect the viability of bacterial cells (Figure 6B,D).

The sensitivity of the *E. meliloti nifA* promoter to IAA and to the gyrase inhibitor novobiocin was evaluated by measuring the relative β-galactosidase activity of bacterial cells, grown aerobically in nitrogen-free medium, after treatment with the two molecules. A significant inhibitory effect on enzyme activity was registered after three and six hours of novobiocin treatment (Figure 6A), suggesting that the promoter of *nifA* was sensitive to changes in DNA topology. An opposite effect was observed when the cells were treated with 1 mM IAA: A significant increase of β-galactosidase activity was measured over time, with the highest induction recorded after 30 min (Figure 6C). These results support the idea that the IAA and novobiocin may regulate *nifA* gene expression, probably by modulating the activity of its promoter through changes in its supercoiling state.

## 4. Discussion

In 1978, Witham [17] hypothesized that the cellular perception and reception of phytohormones could be due to a specific interaction between phytohormones and DNA. Following such a hypothesis, we carried out in vivo and in vitro experiments to verify the existence of a possible connection between IAA action and DNA structure/function. In vivo experiments were carried out with two unrelated bacteria, the soil bacterium poor IAA-producer *E. meliloti* and the enteric bacterium *E. coli,* which does not synthesize IAA, each harboring specific plasmids. The two bacteria were treated with IAA at concentrations not affecting the bacterial growth, and the topological state of the two plasmids DNA was evaluated over time. Interestingly, in both cases, the plasmids from the IAA-treated cells showed an increase of DNA supercoiling in a concentration-dependent manner, suggesting that IAA influences the DNA topological state in vivo. In vitro analysis by CD spectroscopy was then carried out to characterize the IAA–DNA interaction. We demonstrated that IAA triggered conformational changes commonly induced by intercalating agents [45,46]. Moreover, when a competitive study was performed, the addition of increasing concentrations of IAA to the EtBr–ctDNA complex led to a decrease in the binding sites of DNA available for EtBr, indicating that IAA could displace the intercalator EtBr from its EtBr–ctDNA equilibrium complex in a concentration-dependent manner. The effect of IAA on the activity of selected DNA topoisomerase enzymes, whose activity regulates the DNA topological state in bacteria, was then analyzed. The data obtained demonstrated that IAA specifically inhibited the DNA relaxation activity of *EcTopoI* but did not affect the supercoiling activity of DNA gyrase. In our conditions, molecules structurally and functionally similar to IAA did not affect the activity of *EcTopoI*, indicating that the inhibition of the topoisomerase is a peculiarity of IAA (Supplementary Materials Figure S3). We cannot exclude that the indole ring present in the structure of IAA could play an important role in the intercalation process. However, we hypothesize that the mere presence of the indole ring is not sufficient to inhibit the activity of the topoisomerase I enzyme. Therefore, we suggest that three chemical components are important for the interaction of IAA with DNA: The indole ring, the hydroxyl groups available for the formation of hydrogen bonding, and the right distance between the hydroxyl and the indole ring. Another important result is that the inhibition occurred only when the DNA and IAA were pre-incubated in the reaction mixture, demonstrating that the interaction between IAA and DNA is essential for enzyme inhibition. These results provide evidence that the *EcTopoI* inhibition was not due to the direct interference of IAA with the enzyme but to the intercalation of IAA into DNA, preventing the relaxation of the negative supercoiled plasmid by the enzyme. Such results were consistent with the increased negative supercoiling observed in vivo for IAA-treated cells. The overall data suggest that IAA has a role in the modification of DNA supercoiling. Many genes are activated by increasing negative supercoiling, as it favors the unwinding of the DNA double helix required for the formation of open complexes, thus leading to an increase in the transcription rate of promoters for which open complex formation is rate limiting [43,44]. When the mRNA levels of nitrogen fixation and stress related genes were measured in *E. meliloti* 1021 cells treated with IAA and novobiocin, opposite results have been recorded: The expression of these genes was significantly induced in IAA-treated cells and strongly reduced after novobiocin treatment. The highest repressive effect induced by novobiocin was measured for the transcription of the *topA* gene, whose expression is supercoiling sensitive [47].

We also verified, for the first time, that the promoter of *nifA,* the main regulator of nitrogen-fixation genes in *E. meliloti* 1021, was significantly activated in the presence of IAA and inhibited by novobiocin, which reduces the level of bacterial DNA supercoiling. These results suggest that the *nifA* promoter activity is regulated by changes in DNA supercoiling. Such data were consistent with the ones previously obtained from transcriptional and biochemical studies carried out on *E. meliloti* [8]. Our results are also in line with previous data concerning the stimulatory effect of DNA supercoiling on the activity of the *rpoH* promoter in *E. coli* [48], and with the involvement of the heat shock protein encoded by *dnaK* in the maintenance of local DNA supercoiling [49]. The in vivo and in vitro results obtained here for the IAA–DNA interaction suggest a role for IAA in the modulation of DNA topological changes. It is known that different types of DNA-binding agents can act as powerful sequence-specific gene modulators, by exerting their effect from transcription regulation to gene modification [46]. We thus think that a deepening of our results could help to understand whether the new IAA function is involved in promoting the expression of specific genes, including the ones involved in nitrogen fixation.

## 5. Conclusions

Our study demonstrated that IAA is able to influence the DNA topology and expression of genes sensitive to changes in DNA supercoiling. We here speculate that IAA can be considered a signal molecule, able to induce changes in the expression of selected groups of genes involved in various processes, including those related to nitrogen fixation. The intercalation of small molecules into DNA should generally be independent of the base-pair sequence. However, several literature studies concerning the action of anticancer agents, including topoisomerase inhibitors, show that the intercalation of these molecules preferentially occurs at specific sequences [22]. In addition, Witham’s model [17] illustrated a possible in vivo stereochemical recognition between nucleic acids and intercalated phytohormones, including IAA, providing insights as to the specific interactions between phytohormones and DNA. We thus suggest that a specific IAA intercalation could be plausible. We are aware that such major advancements in the knowledge of the IAA action require ulterior strengthening and open further discussions. The specificity of IAA binding at each site and a probable preference for intercalation in specific sequences, at different IAA doses, are just some of the aspects, which still require a far deeper understanding. IAA is a molecule able to affect many cellular processes in unrelated organisms: It acts as classical phytohormone in plants [4]; influences the expression of genes involved in energy metabolism and stress response in soil and enteric bacteria [6,7,8]; and stimulates the transition to filamentous infective *Saccharomyces cerevisiae* in yeast cells [5]. Furthermore, it has been reported that the combination of IAA and horseradish peroxidase [3], or a compound structurally very similar to IAA [50], indole-3-carbinol, could potentially be used for the treatment of human cancer. Overall, the findings discussed here lead to the conclusion that IAA could be more than just a simple plant hormone. 

## Figures and Tables

**Table 1:** Primers used in qRT-PCR analysis.

Gene ID	Gene	Forward (5′→3′)	Reverse (5′→3′)
SMc04040	***ibpA***	GACCTATCCGCCCTACAACA	GAAGCGGACTTGATCTCGAC
SMc00646	***rpoH1***	GTGAGGAAGAGGTCGTCTCG	TCAGAACCTTCATGGCATTG
SMc02163	***pgi***	GGCAAGAAGATCACCGATGT	GTCTCGATCGTGGTGAAGGT
SMa1227	***fixJ***	CTCGTGACGGACCTGAGAAT	GCAGCAACCAGATGTTCAGA
SMa0815	***nifA***	CCTTGCAAGAGCATTCCTTC	TCTTTGACCTGGCGAGAGTT
SMa1225	***fixK1***	CATTCTTTCTTTGCCGAAGC	CGCAAAGATCGACGAGAAAT
SMc02857	***dnaK***	CCGAGTTCAAGAAGGAGCAG	AGCTTCATCGTCAGGTGCTT
SMc01364 SMc02482 SMc02481 SMc02480 SMc00869 SMc00768	***topA*** ***sucA*** ***sucD*** ***icd*** ***atpF2*** ***aceA***	CATCGACCGTGACTATGTGG AAGACCGTCGTCCAGCTCTA TGTTCCAGACGACCAATGAA AACCTGGACGAATCGATCAC GCTGCTTACGAGCAGGAGTT GGACGCTATTCCATCTGGTC	GCACGTCCTTCCAATTGAGT CGACCTCCTTCAACTGCTTC CCTCGTCTTTCAGGAACTGC TTCCTCGTCGAACACCTTCT GCAAGAGCCTTCGACTTGAT CGAGAACGTTGCGATTGTAG
SMc01317	***rpoB***	CGTCAACAAGTACGGCTTCA	CGTCCATCAGGTTGATGTTG

## Supplementary Materials

The following are available online at https://www.mdpi.com/2218-273X/9/10/522/s1, Figure S1. Effect of IAA on DNA topology in *Ensifer meliloti* cells, Figure S2. Effect of IAA on DNA topology in *Escherichia coli* cells, Figure S3. The Inhibition of topoisomerase I from *E. coli* (*EcTopoI*) is a Peculiarity of IAA, Figure S4. IAA does not Exerts an Inhibiting Effect on the Supercoiling Activity of the Type II DNA Gyrase.
