# Supplementary material for: New Insights into Structural and Functional Roles of Indole-3-acetic acid (IAA): Changes in DNA Topology and Gene Expression in Bacteria
Source: Biomolecules. 2019 Sep 23;9(10):522. doi: 10.3390/biom9100522 (PMC6843775; doi:10.3390/biom9100522)
Supplement: Supplementary file 1 [file biomolecules-09-00522-s001.pdf]

Supplementary Materials

New insights into structural and functional roles of Indole-3-acetic acid (IAA): changes in DNA topology and gene expression.

Authors: R. Defez[1,§], A. Valenti[1,§], A. Andreozzi[1], S. Romano[1], M. Ciaramella[1†], P. Pesaresi[2], S. Forlani[2], C. Bianco[1,*]

Affiliation: [1]Institute of Biosciences and BioResources, via P. Castellino 111, 80131 Naples, Italy
[2] Dipartimento di Bioscienze, Università degli Studi di Milano, via Celoria 26, 20133 Milan, Italy
[§] These authors contributed equally to this article.
[†] Deceased on 2 December 2018.

[*]**Corresponding author:**

E-mail: carmen.bianco@ibbr.cnr.it

Telephone number: +39 0816132610

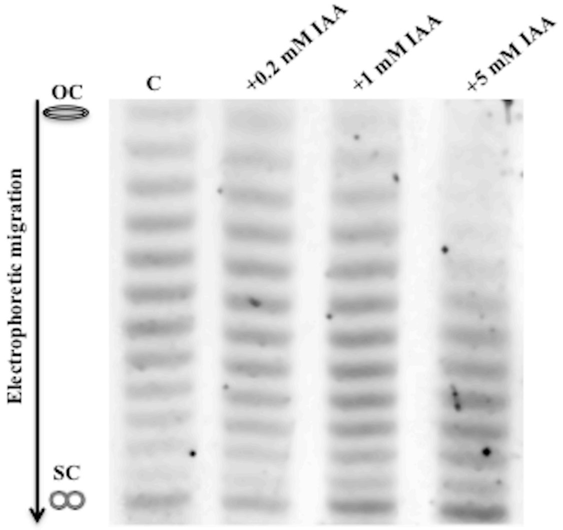

Figure S1. Effect of IAA on DNA topology in *Ensifer meliloti* cells. The pMB393 plasmid was purified from *E. meliloti* 1021 control cells and the ones treated for 60 min with IAA (0.2, 1 and 5 mM) and separated by electrophoresis in 0.7% agarose gels containing 10 μg ml^{-1} chloroquine.

To visualize the reaction products, the gels were stained post-electrophoresis with ethidium bromide and de-stained in deionized water. The relative amount of each topoisomer changed as the concentration of IAA increased. In particular, the topoisomers having high density of negative supercoiling (lower bands) were clearly visible after treatment with 5 mM IAA.. OC, open-circular DNA; SC, supercoiled DNA.

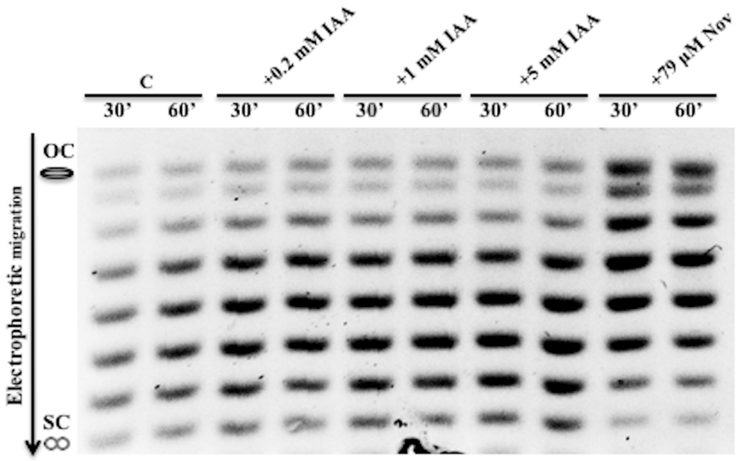

Figure S2. Effect of IAA on DNA topology in *Escherichia coli* cells. The pBS plasmid was purified from *E. coli* BL21 control cells and the ones treated for 30 and 60 min with IAA (0.2, 1 and 5 mM) or Novobiocin (79 µM) and separated by electrophoresis in 0.7% agarose gels containing 1 µg ml^{-1} chloroquine. To visualize the reaction products, the gels were stained post-electrophoresis with ethidium bromide and de-stained in deionized water. The topoisomers generated after IAA-treatment became more negative supercoiled with increasing concentration of IAA. By contrast, the Novobiocin-treatment inhibits DNA gyrase activity and leads, as expected, to a reduction in the negative supercoiling level of plasmids. OC, open-circular DNA; SC, supercoiled DNA.

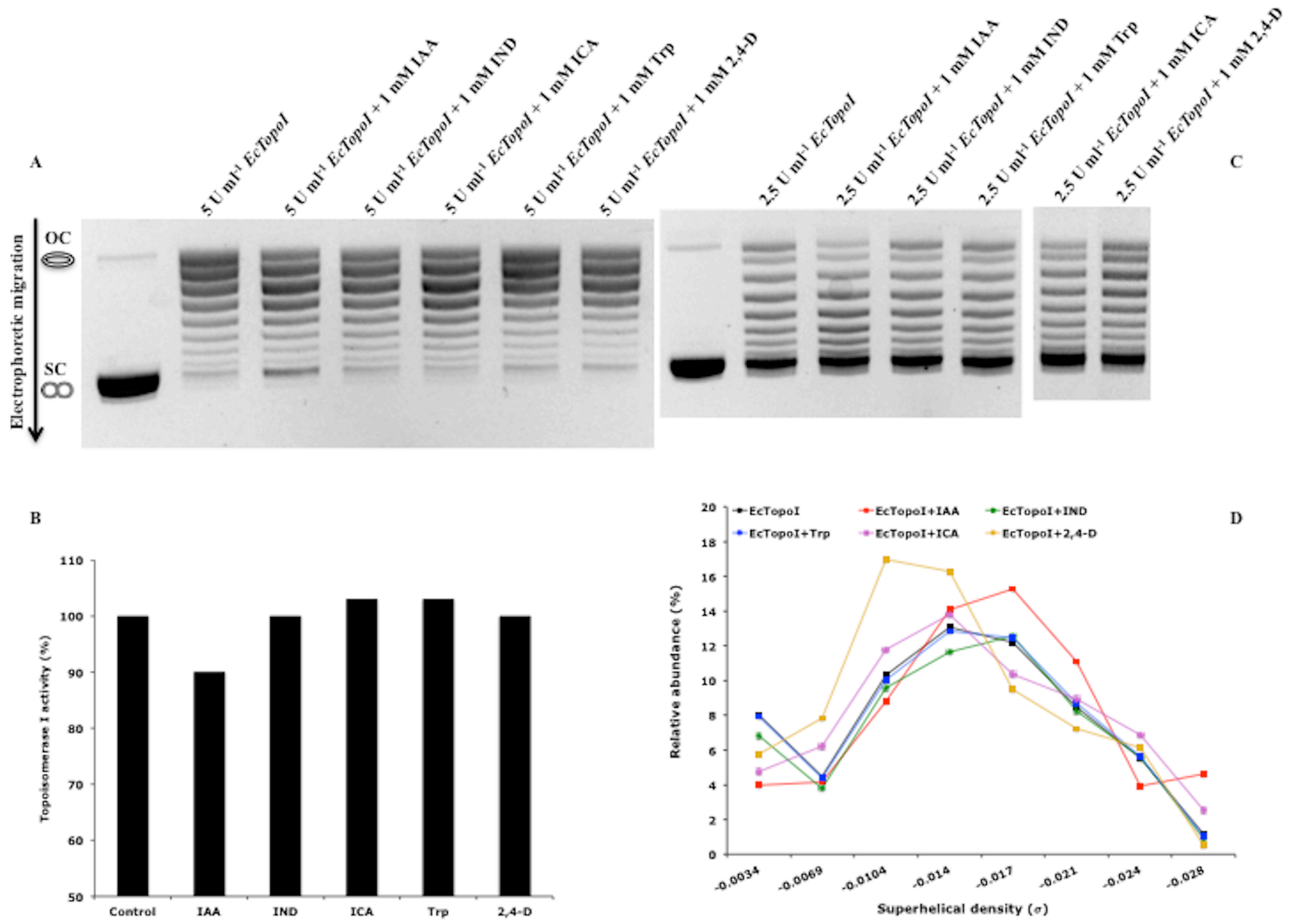

Figure S3. The Inhibition of Topoisomerases is a Peculiarity of IAA. The effect of purified molecules, structurally (indole, IND; indole-3-carboxylic acid, ICA; tryptophan, Trp) or functionally (2,4-dichlorophenoxyacetic acid, 2,4-D) similar to IAA, on the activity of *EcTopoI* was analysed by adding 5 U ml^{-1} (**A** and **B**) and 2.5 U ml^{-1} (**C** and **D**) of enzyme to a mixture containing the supercoiled DNA plasmid and each compounds at 1 mM concentration. The mixtures were then incubated at 37°C for 30 min. Before the addition of the enzyme, the DNA plasmid was incubated with or without the purified molecules for 15 min at room temperature to ensure binding equilibrium. To visualize the reaction products, the gels were stained post-electrophoresis with ethidium bromide and de-stained in deionized water. The amount of all individual topoisomers was expressed as percentage and was calculated by dividing the intensity of each topoisomer band by the amount of total DNA in each lane. In our conditions, none of the compound tested affected the relaxation of supercoiled plasmid DNA in a topoisomerase I-dependent fashion, indicating that the inhibition of topoisomerases is a peculiarity of IAA. OC, open-circular DNA; SC, supercoiled DNA.

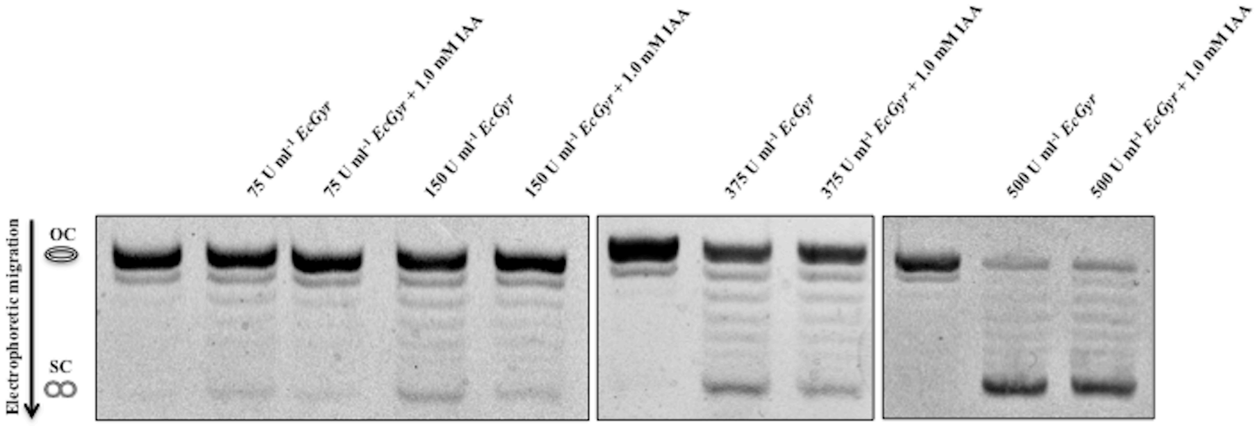

Figure S4. IAA does not exerts an inhibiting effect on the supercoiling activity of the type II DNA gyrase. The effect of IAA on the activity of gyrase was analysed by adding increasing amounts of enzyme (75, 150 and 375 U/ml) to a mixture containing the relaxed DNA plasmid and 1 mM IAA. The mixture was then incubated at 37°C for 30 min. Before the addition of the enzyme, the DNA plasmid was incubated with or without IAA (1mM) for 15 min at room temperature to ensure binding equilibrium. The relaxed DNA plasmid treated with IAA and the relaxed DNA plasmid used as control had been treated similarly. To visualize the reaction products, the gels were stained post-electrophoresis with ethidium bromide and de-stained in deionized water. Under the analysed conditions, IAA did not exert a significant inhibitory effect on the supercoiling activity of the type II DNA gyrase. OC, open-circular DNA; SC, supercoiled DNA.
